# A study on the impact of health shocks on subjective wellbeing of middle-aged people and older adults—Evidence from China

**DOI:** 10.3389/fpubh.2023.1238026

**Published:** 2024-01-11

**Authors:** Qinglin Xu, Jinghong Gu, Cangcang Jia, Huiying Chen, Zihao Li, Hai Gu

**Affiliations:** ^1^Center for Health Policy and Management Studies, School of Government, Nanjing University, Nanjing, China; ^2^Department of Social Science, University of Washington, Seattle, WA, United States; ^3^School of Health Policy and Management, Nanjing Medical University, Nanjing, China

**Keywords:** health shocks, chronic health shocks, acute health shocks, subjective wellbeing, social participation

## Abstract

**Introduction:**

The health issues that afflict middle-aged people and older individuals are a significant factor that affects their quality of life. It is crucial to investigate the impact of health shocks on the subjective wellbeing of this demographic and the mechanisms that underlie this impact to promote healthy aging.

**Methods:**

This study utilized data from the China Family Panel Study in 2018 and 2020 to analyze the effects of HSs and their categories on the subjective wellbeing of middle-aged people and older individuals using the propensity score matching difference-in-differences method. Additionally, the study explored the mediating role of social participation.

**Results:**

The findings indicate that health shocks, both chronic and acute, diminish the subjective wellbeing of middle-aged people and older adults. Furthermore, these shocks have a more significant negative effect on the subjective wellbeing of individuals aged 60 and above, women in the middle-aged and older demographic, individuals in rural areas who belong to the middle-aged and older age groups, and individuals possessing activities of daily living. The mechanism analysis revealed that health shocks, both chronic and acute, reduce the subjective wellbeing of middle-aged people and older individuals by disrupting partnerships.

**Discussion:**

Lowering the possibility of health shocks, the government should build a strong health management system and improve the health insurance system to enable timely treatment for persons suffering from health shocks. Individuals and families should live healthy lives and engage in social activities to avoid health shocks and improve subjective wellbeing.

## 1 Introduction

As per the National Bureau of Statistics of the People's Republic of China, by the end of 2022, 14.9% of China's population will be aged 65 and over (1), marking the country's entry into an aging society. Despite the increasing life expectancy in old age, there has been no corresponding improvement in health functioning (2, 3). The aging of the population has significant implications for economic and social development, with the health status and quality of life of older individuals playing a crucial role (4). The risk of disability and cognitive impairment also increases significantly with age (5, 6). Given this context, promoting healthy aging and enhancing the wellbeing of older individuals has become a pressing concern for both the government and society at large. In 2019, the State Council issued the Opinions on Promoting the Development of Elderly Services, which aims to improve the wellbeing, access, and security of older individuals (7). The international community has also recognized the importance of promoting people's wellbeing, with the United Nations publishing the World Happiness Report annually since 2012 to guide governments and society in focusing on people's happiness. Therefore, studying the factors that influence subjective wellbeing and identifying ways to improve it is crucial for achieving healthy aging and enhancing the quality of life of older individuals (8).

Subjective wellbeing is a crucial psychological indicator of an individual's quality of life, which can be directly influenced by changes in health status (9). The correlation between health status and wellbeing has garnered academic interest, particularly in the context of older individuals. Research suggests that good health can contribute to increased levels of subjective wellbeing in older individuals (10). Conversely, when older individuals experience impaired health, such as reduced perceptual speed or impaired vision and hearing, their subjective wellbeing is also diminished (11, 12). However, it has also been posited that there is no significant effect between the two, and that subjective wellbeing is relatively stable in older individuals with Parkinson's syndrome (13). In the literature, there is little consensus on the association between health and subjective wellbeing in older persons. Furthermore, the mechanisms influencing the association between health shocks and subjective wellbeing have not received much attention in the literature. A person's ability to engage in social activities may be restricted by deteriorating health (14), and social contacts are a crucial source of emotional and supportive resources for people (15). Therefore, further research is needed to ascertain whether a health shock affects an individual's social participation and subsequently influences their subjective wellbeing.

This study aims to objectively measure changes in health among middle-aged people and older adults. By distinguishing between different types of health shocks, examining how they affect the subjective wellbeing of this demographic, and comparing the differences between chronic health shocks and acute health shocks on their subjective wellbeing, we can reveal the pathways through which they experience health shocks and explore strategies to improve their subjective wellbeing. Meanwhile, examining differences in the effects of health shocks on subjective wellbeing across different groups of middle-aged people and older adults not only aids in the identification of potential subgroup effects, but also serves as a foundation for future individualized interventions and policy development. To achieve this, a sample of middle-aged people and older individuals aged 45 and above was selected from the China Family Panel Studies (CFPS) 2018 and 2020 microdata. Firstly, the propensity score matching difference-in-differences (PSM-DID) model was utilized to investigate the impact of health shocks on their subjective wellbeing. Secondly, the mediating effect model was employed to analyze the transmission mechanism between health shocks and subjective wellbeing, and to verify the mediating role of social participation. Finally, this study examines the variability of the impacts of health shocks on subjective wellbeing among groups of varying ages, genders, areas of residence, and activities of daily living (ADL). These assessments aim to give a foundation for decision-making in order to promote the wellbeing of middle-aged people and older adults in the context of aging.

Significant contributions have been made by this study. Firstly, this study differentiates between different forms of health shocks. To assess the variations in the impact of different forms of health shocks, the extended analysis is refined from the dimensions of acute and chronic health shocks. This makes the study's findings more objective and reliable. Secondly, the study has explored the transmission mechanism of health shocks on subjective wellbeing of middle-aged people and older adults based on the social capital theory, providing realistic pathways to enhance their subjective wellbeing and achieve the goal of “active aging”. Thirdly, the study included a sample of middle-aged people to determine the impact of health shocks on the subjective wellbeing of middle-aged people. This will enable the government to advance the policy protection gateway and act with middle-aged people to lessen the likelihood of health shocks in old age. Lastly, the research model and analysis methods used in the study have clearly covered the correlation between the target variables, transmission mechanisms, and the effectiveness of current policies, laying the foundation for the establishment of a systematic, multi-level, and effective protection system for the middle-aged and older adults.

## 2 Literature review

### 2.1 Measurement of health shocks

Health shocks refer to sudden and unexpected deterioration of health conditions that require medical care and may lead to long-term consequences (16). In studies related to health shocks, scholars have primarily measured them in terms of a single dimension, and the existing literature can be divided into two main categories. One category is the acute measurement of health shocks using subjective indicators. The indicator most frequently used by scholars is self-rated health, where individuals make judgments based on their own health status (17, 18). Poorer health is considered a health shock. Data for this indicator are readily available and contain a large amount of information, but there are obvious limitations. Self-rated health is somewhat subjective, and individuals' standards of good or bad health may change over time, or they may even choose to conceal their true health status in order to conform to social expectations or avoid stigma (19).

Another category measures health shocks using objective indicators. Some scholars in health economics use anthropometric indicators, such as changes in body weight and body mass index (BMI), to measure health shocks (20). Although these indicators can compensate for the lack of subjective self-assessment of health, they can be influenced by other environmental factors such as age, gender and ethnicity (21) and do not fully reflect current health status.

Other scholars represent health shocks by using impairments in the ability to care for oneself in daily life, medical expenditures, length of hospital stay, or time spent inactive due to illness to represent health shocks (22–24). Although these metrics are less affected by subjective bias, they have limitations. In order to measure health shocks more objectively, some scholars have judged them by the presence or absence of a certain disease (25, 26). However, no distinction is made between types of illnesses, and it is not possible to compare differences in the impact of health shocks caused by different illnesses.

### 2.2 Health shocks and subjective wellbeing

Subjective wellbeing, a fundamental variable in the economics of happiness, refers to an individual's comprehensive evaluation of their state of life and is a composite reflection of their social functioning and adjustment (27). It has been established that subjective wellbeing can alleviate psychological stress and reduce suicidal ideation (28, 29). Moreover, individuals with a high level of subjective wellbeing tend to live longer, thereby prolonging life to some extent (30). The existing literature on the impact of health on subjective wellbeing focuses on both physical and psychological health.

Individuals in better physical health typically exhibit higher levels of subjective wellbeing (31). In contrast, those with chronic physical illness or severe pain exhibit much lower levels of subjective wellbeing than their physically healthy counterparts (32). It is worth noting that mental health significantly affects the subjective wellbeing of older people (33), even more so than physical health levels (34). Generally, optimistic, positive, and healthy emotions have a positive effect on subjective wellbeing in older people (35), while negative emotions such as anxiety and depression have a negative effect (36). Additionally, health and subjective wellbeing are believed to be causal. Kushlev et al. assessed the association between subjective wellbeing and health behaviors in a broad representative sample of nearly 2.5 million respondents from the USA and found that both life satisfaction and positive affect predicted health behaviors (37). Furrer et al. found that patients with higher subjective wellbeing showed lower pain intensity, and pain intensity was reduced in patients with physical illness or disability by means of enhancing subjective wellbeing, such as positive psychology exercises (38). The worldwide outbreak of the COVID-19 pandemic and the measures taken to prevent and control its spread have had a profound impact on the subjective wellbeing of individuals (9). The research revealed that the COVID-19 pandemic precipitated a substantial decline in outdoor physical endeavors, a surge in the duration of time spent on social media and the internet, a notable reduction in subjective wellbeing, and an escalation in the consumption of fast food (39). During the epidemic, a positive attitude toward risk and death was found to be effective in reducing psychological distress and increasing wellbeing (40).

The timing of life events and the interconnection of persons are emphasized in life course theory (41), and the life course factor has a tremendous impact on an individual's health and wellbeing. The timing element underlines the fact that the identical state changes occur at different times and have different consequences on different people (42). According to research, the subjective wellbeing of older persons varies with age (43, 44). Individuals' beginning disadvantages build over time throughout their lives, resulting to a tendency for systematic divergence of health condition among individuals (45). Disparities in gender, urban/rural residence, and activities of daily living contribute to these drawbacks.

Regarding gender, there is no definitive consensus regarding the subjective wellbeing disparities between females and males (46, 47). The enduring female disadvantage in terms of adverse emotional states and subjective wellbeing leads to a greater prevalence of negative psychological experiences and a diminished sense of subjective wellbeing for women compared to men (48, 49). However, this discrepancy is also attributed to the equitable sharing of total household income between both genders, resulting in roughly comparable levels of wellbeing for both men and women (50). Regarding place of residence, individuals encounter distinct social expectations and possess varying socioeconomic resources, which form the basis for their ability to cope with physical and psychological stress (51). Due to China's longstanding urban-rural divide, substantial disparities exist in the availability of health resources for middle-aged people and older adults residing in urban and rural areas (52). Consequently, when faced with health shocks, the trajectories of subjective wellbeing are theoretically divergent for these two groups (53, 54). In terms of daily mobility, disability serves as a risk factor for numerous chronic ailments such as obesity, osteoporosis, and cardiovascular disease (55–57), and it is more likely to have a detrimental impact on the mental health of the aged (58).

### 2.3 The mediating role of social participation

The theoretical mechanism underlying the influence of social participation as a mediating variable in the pathway of health effects on subjective wellbeing encompasses three fundamental facets.

For starters, the hierarchy of needs is an essential prerequisite and cornerstone for social participation and its impact on older people. According to the theoretical framework of requirements theory, once individuals' basic wants are addressed, they naturally desire to meet higher-level needs, resulting in greater satisfaction (59, 60). However, deteriorated health conditions make it difficult to meet physiological and safety needs, preventing the pursuit of higher-level needs (60). Reduced health status is a barrier to social participation, especially in cases of chronic respiratory ailments, physical and cognitive impairments, or psychological dependence, which are more likely to limit individuals' participation in social activities (14, 61–63).

Secondly, social participation in the form of social activities is a significant way for older people to influence their life. According to social capital theory, social capital refers to an individual's acquisition of the collective resources held by a group, encompassing both the quantity and quality of those resources (64). It signifies an individual's capacity to access limited resources through their involvement in a network or within a broader social structure (65). Interpersonal interactions and social networks are regarded as the fundamental constituents of social capital, as proposed by social capital theory. By fostering and nurturing robust social relationships, individuals can gain access to knowledge, resources, and support, thereby enhancing their quality of life and expanding their opportunities (66). Social participation is a key means of promoting social capital generation. Closer social interactions can give individuals with emotional and social support resources (15). Social interaction has been found to be a mediator of physical activity and to lower frailty, depressive symptoms, and suicide risk in later life (67, 68).

Finally, the ultimate goal and destination of social participation for older persons is active aging. One of the three pillars of achieving positive aging is social participation, which attempts to integrate older persons into society (69). Older persons' social participation can, to some extent, provide economic and emotional exchanges with families, as well as foster peace and wellbeing in intergenerational relationships (70). Social participation is a significant contributor to older persons' mental health (71, 72), and it can effectively limit geriatric social isolation while also increasing geriatric social cohesion (72).

### 2.4 Literature summary

The relationship between health and the subjective wellbeing of older persons has not consistently been addressed in the literature, and there are still gaps. The following three aspects are mostly where this is seen. The first is the method used to gauge health. Studies already published focus more on the relationship between subjective health and subjective wellbeing than health shocks, and there is a dearth of information on how different health shocks affect people differently. Secondly, on the mechanism of the influence of health shocks on the subjective wellbeing of the older adults is limited. Existing research focuses mostly on the direct impact of changes in health status on the subjective wellbeing of the aged, but there is a dearth of study on the process through which it has an influence. Subjective wellbeing is influenced not only by the individual, but also by the social environment. It is vital to investigate the influence mechanism and identify a way for improving the subjective wellbeing of middle-aged people and older persons. Thirdly, existing research concentrates on the older over the age of 60 and ignores the middle-aged population. Individuals' health status in middle age has a significant impact on their health later in life. Focusing on the trajectory of individual health status and subjective wellbeing in middle age can assist in providing tailored policy protection for the middle-aged group and lowering the likelihood of sickness in old age.

This paper endeavors to address gaps in the current research literature by examining the influence of health shocks on the subjective wellbeing of middle-aged people and older adults. The study focuses on the objective health dimension of health shocks and distinguishes between the varying effects of chronic health shocks and acute health shocks. The underlying hypothesis posits that health shocks, both chronic and acute, will adversely affect the subjective wellbeing of middle-aged people and older adults. As a result of experiencing health shocks, this demographic may experience a reduction in their social participation and overall wellbeing. The ultimate goal is that these findings will aid in the development of strategies aimed at enhancing and promoting active aging.

## 3 Methodology

### 3.1 Method

The aim of this study is to examine the impact of health shocks on the subjective wellbeing of middle-aged people and older adults. To address this inquiry, the study employed health shocks as a quasi-natural experiment. The PSM-DID model was utilized to quantify the effect of policy implementation by carefully selecting comparable treatment and comparison groups. For the purpose of this study, the treatment group was composed of middle-aged people and older adults who did not experience either a chronic or an acute health shock in 2018 but encountered either a chronic or an acute health shock in 2020. On the other hand, the control group consisted of middle-aged people and older individuals who did not face any chronic or acute health shocks in both 2018 and 2020. The key concept behind the PSM-DID approach is to reselect the treatment and control group samples to determine, for each middle-aged or older individual who has experienced a health shock, the likelihood of exposure to a health shock in the control group. This approach eliminates selectivity bias and confounding bias that stem from the non-random nature of health shocks. As a result, the rescreened treatment and control groups differ in their levels of subjective wellbeing, except for other characteristic variables (both observable and unobservable variables that remain constant over time, as well as unobservable variables that change synchronously over time), which are as similar as possible, to obtain the net impact of health shocks on subjective wellbeing levels. The corresponding PSM-DID estimators are expressed as follows:


$$\begin{array}{l} \text{AT}{\text{T}}_{\text{PSM}-\text{DID}}=\left[Y_{1}^{T}-Y_{0}^{T}|X,D=1\right]-\left[Y_{1}^{C}-Y_{0}^{C}|X,D=0\right] \end{array}$$

D is a dummy variable for exposure to health shocks (1 is the treatment group, 0 is the control group), T is the treatment group, C is the control group, and *Y*_0_ is the level of subjective wellbeing in the ex-ante group, and *Y*_1_ is the level of wellbeing in the ex-post group. The outcome variables in the PSM-DID approach are no longer cross-sectional data for a particular period, but rather the change in data over a continuous period. Specifically for the questions discussed in this paper, the outcome variable captures the change in subjective wellbeing levels of middle-aged people and older adults between 2018 and 2020.

This study draws on the causal mediation analysis approach constructed by VanderWeele (73) to make causal inferences about the mediating roles of partnership, organizational participation, and religious belief. The approach considers the interaction between exposure and mediators, and the regression model can be expressed as:


$$\begin{array}{c} E\left(M|A=a,C=c\right)=\beta_{0}+\beta_{1}a+\beta_{2}{\;}^{'}c\\ E\left(Y|A=a,M=m,C=c\right)=\theta_{0}+\theta_{1}a+\theta_{2}m+\theta_{3}am+\theta_{4}{\;}^{'}c \end{array}$$

The controlled direct effect (CDE), natural direct effect (NDE), and natural indirect effect (NIE) in the above model, as the exposure level changes from a^*^ to *a*, can be estimated as follows:


$$\begin{array}{lll} CDE\left(m\right) & = & \left(\theta_{1}+\theta_{3}m\right)\left(a-a^{*}\right)\\ NDE & = & \left(\theta_{1}+\theta_{3}\beta_{0}+\theta_{3}\beta_{1}a^{*}+\theta_{3}\beta_{2}^{{\;}^{\prime }}\right)\left(a-a^{*}\right)\\ NIE & = & \left(\theta_{2}\beta_{1}+\theta_{3}\beta_{1}a\right)\left(a-a^{*}\right) \end{array}$$

In this study exposure A is the health shock, the two exposure levels *a* = 1 and a^*^ = 0, mediator M is partnership, organizational participation, and religious belief, respectively, Y is the subjective wellbeing of middle-aged and older adults, and C is each type of covariate. The control direct effect [CDE (m)] indicates the average degree of change in subjective wellbeing if the mediator is fixed uniformly at level m in the population, but the health shock is changed from level a^*^ = 0 to level *a* = 1. The natural direct effect indicates how much the outcome would change if the level of exposure was set at *a* = 1 instead of a^*^ = 0, but for each individual the mediator was held at the level it might have taken for that individual in the absence of exposure. The natural indirect effect shows the effect of X on Y through M if the exposure level is fixed at *a* = 1. The above effects are conditional on the level of the covariate *C* = *c*.

### 3.2 Variable selection

#### 3.2.1 Dependent variable

Subjective wellbeing, a psychological state of contentment and pleasure, is a comprehensive cognitive evaluation of one's current quality of life and an overall subjective feedback of one's inner mental state. The variable is measured by the question “Are you happy?” and the scores are categorized into five distinct groups. A score of “0, 1, 2” is attributed a value of “1”, whereas a score of “3, 4” is attributed a value of “2”. A score of “5, 6” is attributed a value of “3”, “7, 8” is attributed a value of “4”, and “9, 10” is attributed a value of “5”. The range of values spans from 1 to 5.

#### 3.2.2 Independent variable

Health shocks. This study measures health shocks in two dimensions, chronic and acute. Chronic health shocks are measured using the question “During the past 6 months, have you had any doctor-diagnosed chronic disease?”, with a “yes” answer assigned to 1 and a “no” answer assigned to 0. Acute health shocks are measured using the question “In the past year, were you ever been hospitalized due to illness”, with a “yes” answer being assigned a value of 1 and a “no” answer being assigned a value of 0. Health shocks are constructed from both chronic health shocks and acute health shocks, and are assigned a value of 1 if the respondent has experienced at least one chronic or acute health shock, and 0 otherwise.

#### 3.2.3 Mediating variable

Social participation. Based on previous research (74), three social participation dimensions—partnership, religious belief, and organizational participation—were chosen for this study. Partnership is measured by “Do you think you are popular?”, with a score of 0 being the lowest and 10 being the highest. Religious belief is measured by “Are you the member of religious group?”, with a value of 1 for a “yes” answer and 0 for a “no” answer. Organizational participation is measured using the questions “Are you the member of Communist Party of China?”, “Are you the member of Labor union?” and “Are you the member of Association of individual workers?”. Organizational participation is assigned a value of 1 if one of the three questions is answered “yes”, and 0 if all three questions are answered “no”.

#### 3.2.4 Control variables

To enhance the accuracy of examining the impact of health shocks on the subjective wellbeing of the older and middle-aged adults, and drawing from relevant research, the variables employed for control primarily encompassed demographic factors, including age, gender, education, marriage, household registration, and residence, socio-economic factors such as medical insurance, pension insurance, self-reported economic status, self-reported social status, physical health factors such as BMI and abilities in daily life (ADL), and lifestyle habits, such as smoking, drinking, exercise, siesta, and internet use. Among them, BMI is classified as underweight (BMI < 18.5), normal weight (18.5 ≤ BMI < 24.0), overweight (24.0 ≤ BMI < 28.0), and obese (BMI ≥ 28.0) according to the Health Industry Standard of the People's Republic of China, WS/T428-2013 Adult Weight Determination. The ability to perform the seven activities of daily living is based on the independent completion of outdoor activities, eating, kitchen activities, using public transport, shopping, cleaning and sanitation, and laundry, with a value of 1 if all seven activities can be completed independently and 0 otherwise.

### 3.3 Dataset

CFPS data from 2018 and 2020 are used in this study. An extensive, nationwide, interdisciplinary survey that focuses on Chinese households' current circumstances and changes has been conducted. The CFPS, which is supported by Peking University and the National Natural Science Foundation of China (NSF), conducts research on a wide range of subjects, including family dynamics, social engagement, education, job, migration, and health. To more accurately portray Chinese society, the CFPS employs implicit stratification and multistage probability sampling proportional to size (75). The CFPS baseline survey sample was drawn in three stages: county, village, and household, and it covered 25 provinces and districts in mainland China and 95% of the population (76). This gives us a more representative sample to investigate the influence of health shocks on the subjective wellbeing of middle-aged people and older persons. After subtracting the missing values of key variables, not applicable values, “unable to judge,” “refused to answer,” “don't know,” and “situation not applicable” from the sample, the final number of valid samples was 8,296. The sample sizes for 2018 and 2020 are both 4,148, respectively, out of the total sample. In Table 1, we provide the sample sizes for the control and treatment groups in the two data periods.

**Table 1:** Comparison of relevant variables between the treatment and control groups.

**Variables**	**2018**							**2020**						
	**T**	**C**						**T**	**C**					
		**Health shocks**		**Chronic health shocks**		**Acute health shocks**			**Health shocks**		**Chronic health shocks**		**Acute health shocks**	
	**Mean**	**Mean**	**Mean diff**.	**Mean**	**Mean diff**.	**Mean**	**Mean diff**.	**Mean**	**Mean**	**Mean diff**.	**Mean**	**Mean diff**.	**Mean**	**Mean diff**.
Subjective wellbeing	3.992	3.788	0.204^***^	3.776	0.216^***^	3.813	0.179^**^	4.008	3.519	0.489^***^	3.531	0.476^***^	3.493	0.515^***^
Partnership	7.258	7.060	0.199^**^	7.063	0.195^*^	7.053	0.205	7.239	6.728	0.511^***^	6.743	0.497^***^	6.700	0.539^***^
Religious belief	0.035	0.040	−0.004	0.043	−0.007	0.033	0.002	0.03	0.038	−0.008	0.043	−0.013	0.027	0.003
Organizational participation	0.099	0.079	0.019	0.079	0.019	0.080	0.019	0.116	0.075	0.041^***^	0.073	0.043^**^	0.080	0.036
Age														
45–50	0.699	0.618	0.081^***^	0.624	0.076^***^	0.607	0.093^**^	0.654	0.545	0.108^***^	0.551	0.102^***^	0.533	0.120^***^
60 years and above	0.301	0.382	−0.081^***^	0.376	−0.076^***^	0.393	−0.093^**^	0.346	0.455	−0.108^***^	0.449	−0.102^***^	0.467	−0.120^***^
Gender (1 = male)	0.524	0.439	0.085^***^	0.426	0.099^***^	0.467	0.058	0.524	0.437	0.087^***^	0.422	0.102^***^	0.467	0.058
Education														
Illiterate/semi-literate (1 = yes)	0.286	0.353	−0.067^***^	0.350	−0.064^**^	0.360	−0.074^**^	0.286	0.353	−0.068^***^	0.350	−0.064^**^	0.360	−0.074^**^
Primary school (1 = yes)	0.288	0.256	0.032	0.248	0.041	0.273	0.015	0.288	0.256	0.032	0.248	0.041	0.273	0.015
Lower secondary (1 = yes)	0.302	0.272	0.031	0.290	0.012	0.233	0.069^*^	0.302	0.272	0.031	0.29	0.012	0.233	0.069^*^
High school and above (1 = yes)	0.124	0.119	0.004	0.112	0.011	0.133	−0.01	0.124	0.119	0.005	0.112	0.012	0.133	−0.009
Household registration (1 = non-farm)	0.168	0.148	0.020	0.145	0.023	0.153	0.015	0.158	0.139	0.019	0.132	0.026	0.153	0.005
Marriage	0.924	0.912	0.013	0.898	0.027^*^	0.940	−0.016	0.914	0.887	0.027^*^	0.881	0.033^*^	0.900	0.014
Residence	0.417	0.406	0.010	0.406	0.011	0.407	0.010	0.423	0.406	0.017	0.399	0.023	0.420	0.003
Medical insurance	0.936	0.932	0.004	0.921	0.015	0.953	−0.017	0.874	0.879	−0.005	0.871	0.002	0.893	−0.020
Pension insurance	0.545	0.494	0.051^**^	0.502	0.044	0.480	0.065	0.501	0.43	0.070^***^	0.442	0.059^**^	0.407	0.094^**^
Self-reported economic status	3.022	2.832	0.190^***^	2.825	0.197^***^	2.847	0.175^*^	3.118	2.784	0.334^***^	2.752	0.366^***^	2.847	0.271^***^
Self-reported social status	3.257	3.130	0.127^**^	3.096	0.161^**^	3.200	0.057	3.348	3.049	0.299^***^	3.02	0.328^***^	3.107	0.241^***^
BMI														
Underweight (1 = yes)	0.048	0.057	−0.009	0.056	−0.008	0.060	−0.012	0.043	0.064	−0.021^**^	0.053	−0.010	0.087	−0.044^**^
Normal weight (1 = yes)	0.547	0.547	0.000	0.538	0.009	0.567	−0.019	0.55	0.532	0.018	0.545	0.005	0.507	0.043
Overweight (1 = yes)	0.323	0.305	0.018	0.323	−0.001	0.267	0.056	0.329	0.311	0.017	0.310	0.018	0.313	0.015
Obese (1 = yes)	0.082	0.091	−0.009	0.083	−0.001	0.107	−0.025	0.078	0.093	−0.014	0.092	−0.014	0.093	−0.015
ADL	0.985	0.974	0.012^*^	0.967	0.018^**^	0.987	−0.002	0.766	0.687	0.079^***^	0.693	0.073^***^	0.673	0.092^***^
Smoking	0.347	0.294	0.053^**^	0.284	0.063^**^	0.313	0.033	0.329	0.276	0.053^**^	0.281	0.049^*^	0.267	0.062
Drinking	0.201	0.170	0.031	0.188	0.013	0.133	0.067^**^	0.187	0.139	0.048^**^	0.152	0.035	0.113	0.074^**^
Exercise	0.445	0.459	−0.014	0.432	0.013	0.513	−0.068^*^	1.000	1.000	0.000	1.000	0.000	1.000	0.000
Siesta	0.564	0.585	−0.021	0.578	−0.014	0.600	−0.036	0.639	0.687	−0.048^**^	0.693	−0.054^*^	0.673	−0.034
Internet use	0.311	0.329	−0.018	0.330	−0.019	0.327	−0.016	0.386	0.366	0.020	0.373	0.014	0.353	0.033
*N*	3695	453		303		150		3695	453		303		150	

^*^, ^**^, and ^***^ denote 10%, 5%, and 1% levels of significance respectively.

Table 1 displays the means and mean differences for all variables before and after health shocks for both treatment and control group samples. As shown in the table, the difference in mean values of subjective wellbeing between the treatment and control groups was 0.204, 0.216, and 0.179 before exposure to health shocks, chronic health shocks, and acute health shocks, respectively, and the difference in both increased after the experience. Acute health shocks generated the biggest difference of 0.515.

## 4 Results

### 4.1 Baseline regression

#### 4.1.1 Propensity score matching logit estimation

The first step in using the PSM-DID method is to use the logit model to match the samples of the treatment and control groups in the base period. By controlling for the covariates affecting middle-aged people and older adults, the probability of being exposed to health shocks in the treatment and control groups after the matching is completed is similar to avoid biased estimates due to sample selectivity bias. Tables A1–A3 present the results of the propensity score estimates and balance tests for health shocks, chronic health shocks, and acute health shocks, respectively.

#### 4.1.2 Balance test

Passing the balance test is required before using PSM-DID. According to the balance test results (Tables A1–A3), there were significant differences in the means of the characteristic variables between the treatment and control groups before matching, but the systematic deviations of the characteristic variables were all < 10% after matching. Except for the medical insurance variable in Table A1, which no longer differed systematically between the treatment and control groups after matching, all variables passed the test.

To ensure matching quality, the PSM method is only valid in the common support domain. Therefore, before formally estimating the mean treatment effect, the common support hypothesis also needs to be tested to ensure that propensity scores have a sufficient number of overlapping regions in the treatment and control groups. Figure A1 illustrates the distribution of individual propensity scores for the health shock, chronic health shock, and acute health shock treatment and control group samples as well as areas of common support. Kernel density plots of propensity scores before and after matching are shown in Figures A2–A4. The treated and control groups' kernel density trends after matching are essentially the same and have a high degree of overlap compared to the pre-matching, showing that the matching findings are excellent.

#### 4.1.3 PSM-DID analysis results

The balance test analysis shows that the PSM results are valid, thus allowing for double-differencing using the successfully matched samples. The kernel matching method was used for estimation in this paper and the results are presented in Table 2. The results demonstrate that health shocks diminish the levels of subjective wellbeing among individuals in the middle-aged and older adult cohorts. Specifically, the impact of health shocks is estimated to be a reduction of 7.4% (−0.282/3.788 × 100%) in subjective wellbeing levels. In the case of chronic health shocks, the decrease is estimated at 6.9% (−0.260/3.776 × 100%), while acute health shocks result in an 8.7% (−0.332/3.813 × 100%) decline in subjective wellbeing levels for middle-aged individuals and older adults. This is evident in the more severe negative consequences of acute health shocks.

**Table 2:** Results of PSM-DID estimation of health shocks on subjective wellbeing of middle-aged people and older adults.

**Variables**		**Health shocks**	**Chronic health shocks**	**Acute health shocks**
Before	C	3.976	3.977	3.993
	T	3.785	3.776	3.812
	Diff. (T-C)	−0.191^***^ (0.028)	−0.202^***^ (0.029)	−0.181^***^ (0.031)
After	C	3.990	3.993	4.003
	T	3.518	3.531	3.490
	Diff. (T-C)	−0.473^***^ (0.028)	−0.461^***^ (0.029)	−0.514^***^ (0.031)
Diff-in-diff		−0.282^***^ (0.040)	−0.260^***^ (0.040)	−0.332^***^ (0.043)
Control variables		Yes		

^*^, ^**^, and ^***^ denote 10%, 5%, and 1% levels of significance respectively.

### 4.2 Robustness check

#### 4.2.1 Placebo test

To exclude the effects of omitted variables and potentially unobservable factors, reference was made to Chetty et al. (77), where a random sample of health shocks and subjective wellbeing were randomly selected from the total sample using the Bootstrap method to conduct a placebo test. To ensure the reliability of the estimation results, 1,000 regressions were conducted using the baseline model. According to the robustness test criteria, when the true estimated coefficients deviate from the estimated coefficients of the random sample, the benchmark results are considered to be free from model setting bias, unaffected by the interference of omitted variables, and robust. Figure A5 reports the distribution of the estimated coefficients. The coefficients obtained from the random sample estimation are all distributed around 0, indicating that the estimation results of the baseline model in this study are not affected by the interference of omitted variables.

#### 4.2.2 Adjusting the treatment of the dependent variable

In the previous estimation, the dummy variable “subjective wellbeing” was treated as a five-category variable. This paper uses the questionnaire's original subjective wellbeing measure, or the eleven-category classification, to test the results' reliability. Table 3 displays the findings of the regression analysis of the explanatory factors for the eleven categorizations. The regression coefficients are still significant even when the explanatory variables have been treated differently, and the conclusion that acute, chronic, and health shocks all lower subjective wellbeing in middle-aged people and older persons is strong.

**Table 3:** Robustness test results.

**Variables**		**Adjusting the treatment of subjective wellbeing**			**Substitution of dependent variables**		
		**Health shocks**	**Chronic health shocks**	**Acute health shocks**	**Health shocks**	**Chronic health shocks**	**Acute health shocks**
Before	C	7.569	7.572	7.608	4.083	4.087	4.108
	T	7.122	7.099	7.188	3.863	3.795	4.007
	Diff. (T-C)	−0.447^***^ (0.064)	−0.473^***^ (0.065)	−0.420 (0.068)	−0.220^***^ (0.029)	−0.291^***^ (0.030)	−0.101^***^ (0.030)
After	C	7.607	7.611	7.636	4.158	4.159	4.176
	T	6.496	6.551	6.383	3.490	3.673	3.725
	Diff. (T-C)	−1.111^***^ (0.064)	−1.060^***^ (0.065)	−1.254^***^ (0.068)	−0.467^***^ (0.029)	−0.486^***^ (0.030)	−0.451^***^ (0.030)
Diff-in-diff		−0.664^***^ (0.091)	−0.587^***^ (0.092)	−0.834^***^ (0.096)	−0.248^***^ (0.041)	−0.195^***^ (0.042)	−0.350^***^ (0.042)
Control variables		Yes					

^*^, ^**^, and ^***^ denote 10%, 5%, and 1% levels of significance respectively.

#### 4.2.3 Substitution of the dependent variable

Subjective wellbeing refers to an individual's personal evaluation of their current mental and life quality. It is closely related to life satisfaction, as those with elevated subjective wellbeing tend to have higher levels of life satisfaction. Hence, in this study, the life satisfaction measure was chosen as a robustness check. The regression outcomes, displayed in Table 3, demonstrate that even when substituting the dependent variable, health shocks, chronic health shocks, and acute health shocks continue to diminish the subjective wellbeing of middle-aged people and older individuals.

### 4.3 Heterogeneity

Based on life course theory, this study analyzed heterogeneity in four dimensions: age, gender, urban/rural and ability to perform daily activities factors. According to the findings in Table 4, four groups—people over the age of 60, women in middle and later life, rural middle-aged and senior citizens, and middle-aged and older individuals who can perform daily tasks—are more negatively impacted by health shocks, chronic health shocks, and acute health shocks.

**Table 4:** Results of heterogeneity analysis.

			**Age**		**Gender**		**Residence**		**ADL**	
			**45–59**	**60 years and above**	**Male**	**Female**	**Urban**	**Rural**	**Normal**	**Disabled**
Health shocks	Before	C	3.940	4.050	3.947	4.006	4.046	3.920	3.979	3.457
		T	3.718	3.907	3.795	3.802	3.815	3.770	3.789	3.667
		Diff. (T-C)	−0.221^***^ (0.034)	−0.143^***^ (0.051)	−0.152^***^ (0.038)	−0.203^***^ (0.043)	−0.230^***^ (0.043)	−0.150^***^ (0.038)	−0.191^***^ (0.028)	0.210 (0.475)
	After	C	3.944	4.065	3.982	3.999	4.009	3.981	4.002	3.077
		T	3.465	3.599	3.603	3.458	3.597	3.464	3.512	4.000
		Diff. (T-C)	−0.478^***^ (0.036)	−0.466^***^ (0.051)	−0.379^***^ (0.038)	−0.541^***^ (0.043)	−0.412^***^ (0.044)	−0.518^***^ (0.038)	−0.490^***^ (0.033)	0.923 (0.964)
	Diff-in-diff		−0.257^***^ (0.050)	−0.324^***^ (0.072)	−0.226^***^ (0.053)	−0.337^***^ (0.060)	−0.182^***^ (0.062)	−0.368^***^ (0.054)	−0.300^***^ (0.043)	0.713 (1.075)
Chronic health shocks	Before	C	3.941	4.041	3.934	4.006	4.039	3.917	3.960	3.719
		T	3.725	3.860	3.744	3.803	3.779	3.772	3.750	3.500
		Diff. (T-C)	−0.216^***^ (0.034)	−0.182^***^ (0.052)	−0.190^***^ (0.038)	−0.203^***^ (0.043)	−0.261^***^ (0.044)	−0.145^***^ (0.038)	−0.210^***^ (0.033)	−0.219 (0.164)
	After	C	3.944	4.069	3.978	4.005	4.002	3.980	4.000	3.757
		T	3.527	3.570	3.594	3.491	3.539	3.514	3.510	3.750
		Diff. (T-C)	−0.417^***^ (0.036)	−0.499^***^ (0.052)	−0.384^***^ (0.038)	−0.513^***^ (0.043)	−0.463^***^ (0.045)	−0.466^***^ (0.039)	−0.491^***^ (0.033)	−0.007 (0.164)
	Diff-in-diff		−0.201^***^ (0.050)	−0.318^***^ (0.074)	−0.194^***^ (0.054)	−0.311^***^ (0.061)	−0.202^***^ (0.063)	−0.321^***^ (0.054)	−0.280^***^ (0.047)	0.213 (0.232)
Acute health shocks	Before	C	3.938	4.081	4.081	4.097	4.184	4.030	4.088	3.989
		T	3.663	4.000	3.967	4.154	3.950	4.105	4.039	4.000
		Diff. (T-C)	−0.275^***^ (0.039)	−0.081 (0.053)	−0.115 (0.075)	0.057 (0.086)	−0.234^***^ (0.089)	0.075 (0.068)	−0.049 (0.053)	0.011 (0.216)
	After	C	3.954	4.089	4.055	4.131	4.148	4.072	4.111	3.817
		T	3.337	3.661	3.633	3.692	3.850	3.595	3.857	3.750
		Diff. (T-C)	−0.616^***^ (0.039)	−0.428^***^ (0.053)	−0.421^***^ (0.075)	−0.439^***^ (0.086)	−0.298^***^ (0.090)	−0.477^***^ (0.068)	−0.254^***^ (0.068)	−0.067 (0.252)
	Diff-in-diff		−0.341^***^ (0.054)	−0.347^***^ (0.075)	−0.306^***^ (0.106)	−0.496^***^ (0.121)	−0.063 (0.126)	−0.553^***^ (0.096)	−0.206^**^ (0.086)	−0.078 (0.332)
Control variables			Yes							

^*^, ^**^, and ^***^ denote 10%, 5%, and 1% levels of significance respectively.

### 4.4 Mechanism

For testing the mechanism, this study employs the causal mediation method (73). Table 5 lists the total effect, natural direct effect, and natural indirect effect of various health shocks on the mediating variables. According to the table, health shocks, chronic health shocks, and acute health shocks all undermine the partnership, lowering the subjective wellbeing of the middle-aged and older persons. And the mediating effects of religious belief and organizational participation are not significant. In addition, Table A4 reports the results of regressions that hypothesize an interaction between the treatment and the mediating variable, and it can be seen that the interaction term between different types of health shocks and the mediating variable has no effect on subjective wellbeing.

**Table 5:** Results of the mechanism analysis.

	**Health shocks**			**Chronic health shocks**			**Acute health shocks**		
	**Total effect**	**Natural direct effect**	**Natural indirect effect**	**Total effect**	**Natural direct effect**	**Natural indirect effect**	**Total effect**	**Natural direct effect**	**Natural indirect effect**
Partnership	−0.291^***^ (0.031)	−0.244^***^ (0.028)	−0.047^***^ (0.131)	−0.287^***^ (0.036)	−0.249^***^ (0.034)	−0.038^**^ (0.014)	−0.303^***^ (0.054)	−0.231^***^ (0.047)	−0.072^**^ (0.028)
Religious belief	−0.291^***^ (0.059)	−0.290^***^ (0.031)	−0.001 (0.001)	−0.283^**^ (0.092)	−0.282^***^ (0.037)	−0.001 (0.002)	−0.307^***^ (0.062)	−0.306^***^ (0.051)	−0.001 (0.001)
Organizational participation	−0.291^***^ (0.043)	−0.292^***^ (0.031)	0.001 (0.002)	−0.283^***^ (0.037)	−0.284^***^ (0.037)	0.001 (0.002)	−0.304^**^ (0.102)	−0.309^***^ (0.051)	0.005 (0.005)
Control variables	Yes								

^*^, ^**^, and ^***^ denote 10%, 5%, and 1% levels of significance respectively.

## 5 Discussion

Subjective wellbeing refers to the evaluation and assessment of one's own life (78), including reflective cognitive judgments such as life satisfaction, as well as emotional reactions to one's current life. Health status is a crucial factor affecting an individual's mood, particularly for middle-aged people and older individuals. In the field of economics, scholars have mainly focused on the relationship between income and wellbeing, with less research conducted on the impact of health shocks on the subjective wellbeing of the middle-aged and older individuals, particularly the differential impact of different types of health shocks. This study evaluates the effects and mechanisms of health shocks on the subjective wellbeing of middle-aged people and older individuals based on nationwide survey data, distinguishing between the effects of chronic health shocks and acute health shocks. Furthermore, the study analyses the heterogeneity of this impact effect across age, gender, and residence groups of middle-aged people and older adults.

The results suggest that health shocks significantly reduce subjective wellbeing in middle-aged people and older adults. Health shocks can reduce the quality of life of middle-aged people and older individuals, as well as the onset of negative emotions such as worry and sadness (79), which ultimately damage their subjective wellbeing. This paper's study interval encompasses the year 2020. Individuals who are infected with COVID-19 during this time may experience a more pronounced drop in subjective wellbeing (80). On the one hand, this is due to the COVID-19 pandemic, which surely caused a broad sense of anxiety, fear, uncertainty, and insecurity (81, 82). On the other hand, were implemented in response to COVID-19, resulting in a considerable drop in medical visits during the lockdown (83). Because of this, patients are unable to receive timely medical care, which may cause their sickness to worsen and impair their subjective feeling of wellbeing.

This study presents a novel approach by distinguishing between health shocks and examining the differences in subjective wellbeing between chronic health shocks and acute health shocks in middle-aged people and older adults. The results demonstrate that acute health shocks have a more pronounced negative impact on the subjective wellbeing of middle-aged people and older adults compared to chronic health shocks. This may be attributed to the more severe impact of acute health shocks on individuals and families, leading to reduced labor supply (84) and increased caregiving burdens on spouses or other family members, ultimately reducing leisure time (85). Such circumstances can cause feelings of guilt toward spouses and lead to lower levels of subjective wellbeing. In contrast, the weaker effect of chronic health shocks on the subjective wellbeing of middle-aged people and older adults may be attributed to the cumulative effect of chronic illness, with negative emotions becoming more prominent over time as the individual becomes chronically ill. Additionally, the number and type of chronic illnesses may also play a crucial role in the subjective wellbeing of middle-aged people and older adults. Multiple chronic illnesses increase the likelihood of hospitalization and reduce the quality of life (86, 87), whereas having only a single chronic illness has a less significant impact on subjective wellbeing.

The impact of health shocks on subjective wellbeing also varies among different groups. Regarding age, the decline in subjective wellbeing is more pronounced in older individuals over 60 years of age when they experience health shocks, chronic health shocks, and acute health shocks. This may be due to the fact that older individuals have reduced functionality compared to middle-aged individuals, exhibit more severe symptoms of illness, and recover more slowly from acute health shocks and chronic health shocks, leading to greater impairment in subjective wellbeing. In terms of gender, the decline in subjective wellbeing is more pronounced in women when they experience health shocks, chronic health shocks, and acute health shocks. Gender inequality has been studied as a form of injustice that can reduce public wellbeing (88). Women are relatively disadvantaged in terms of educational opportunities, division of labor, interpersonal network construction, and access to socio-economic resources due to traditional gender roles (89). These resources can mitigate the adverse effects of health shocks. Women are also more sensitive to stress and emotional expressions in response to negative events (90). Therefore, experiencing a negative event such as health shocks can have a more detrimental effect on the subjective wellbeing of female middle-aged individuals and older adults. As for the place of residence, the reduction in subjective wellbeing levels was more profound for middle-aged people and older individuals residing in rural areas when confronted with health shocks, whether chronic or acute. A plausible explanation for this phenomenon is that the disparity in socio-economic status impedes rural and urban middle-aged people and older individuals from enjoying equal social welfare benefits. In comparison to their urban counterparts, rural middle-aged individuals and older people are subject to inequitable policies regarding fundamental education, health care coverage, and labor market returns. Hence, when exposed to health shocks, rural middle-aged people and older individuals undergo a greater loss of subjective wellbeing due to the urban-rural differences in financial capability, public service provision, and social resource allocation. In terms of activities to daily living, health shocks, chronic health shocks, and acute health shocks have little effect on subjective wellbeing in people with impairments. As the “disability paradox” reveals (91), individuals with severe disabilities are more likely to report a higher quality of life (92, 93). Impaired persons have long evolved to coexisting with their sickness and may actively develop future representations congruent with natural decline and loss (94). Once these declines and losses occur, these expectations may have a less unfavorable psychological impact (95).

The analysis of mediating effects has revealed that partnership mediates the pathways of health shocks, chronic health shocks, and acute health shocks on the subjective wellbeing of middle-aged individuals and older adults. Health shocks cause illness distress for this demographic, which inevitably reduces their social participation. Social participation refers to a multifaceted and deep interaction with others in a society or community (96), and is a positive predictor of subjective wellbeing (97). Illness restricts an individual's interpersonal activities, diminishes the frequency of interactions with friends, and impairs partnerships. Reduced social connectedness leads to a failure to meet the spiritual demands of middle-aged and older persons (98), lowering their subjective wellbeing. One possible reason for the minor mediation impact of organizational participation and religious belief in the Chinese context is that the three types of organizations and religious groups discussed below are formal organizations. Respondents' engagement in these activities, influenced by traditional Chinese culture, implies good status symbols and hence higher motivation. As a result, the effect of health shocks on them is negligible, and hence on subjective wellbeing.

Compared to previous studies, this study has two distinctive features. Firstly, the data for this study were drawn from the CFPS, which is a well-represented national database. Secondly, the study used the PSM-DID methodology to assess the causal relationship between Health shocks and the subjective wellbeing of middle-aged individuals and older adults. This approach effectively eliminates the influence of external confounders on the assessment.

Furthermore, it is crucial to acknowledge certain limitations inherent in this study. The first pertains to the identification of individuals with chronic conditions. While respondents who experienced a chronic health shock during t0 have been excluded from the study to mitigate the inclusion of individuals who initially had a chronic disease in the control group, the available data only ensures that respondents had not received a physician's diagnosis of a chronic condition within the past 6 months at the time of the survey. This data collection, confined to a specific temporal window, fails to capture the existence of underlying chronic conditions at other junctures. Consequently, it may not accurately identify instances where a chronic condition went undiagnosed at time t0 but may indeed be chronic in nature. When this group is incorporated into the control group, they may exhibit lower subjective wellbeing at t1 compared to the control sample without a chronic condition, thereby underestimating the adverse impact of chronic health shocks on subjective wellbeing. In future studies, it would be prudent to contemplate collecting primary data and employing more comprehensive and diversified measures of chronic diseases to comprehensively identify potential individuals with chronic conditions, thereby enhancing the scientific rigor of our research.

Secondly, with regard to the disparity in subjective wellbeing between the middle-aged and older cohorts, the measurement questions pertaining to chronic health shocks and acute health shocks in the CFPS questionnaire solely ascertain whether the sample experienced a negative health event or not. However, these questions do not account for the intensity or magnitude of chronic health shocks or acute health shocks. Regrettably, this limitation impedes our ability to measure whether the divergence in subjective wellbeing between the middle-aged and older adults is attributable to varying degrees of chronic and acute health shocks. In future studies, it may be worthwhile to consider collecting additional data on the strength of chronic and acute health shocks to more effectively discern the underlying factors contributing to the differences in subjective wellbeing between the middle-aged and the older.

Third, there are common causes of worsening in health and spousal fatalities that can be significantly age-graded. However, due to the limitations of the study data, the sample size of older persons aged 75 and up in the study sample was modest. As a result, this study was unable to investigate the variations in subjective wellbeing between the young old (60–74 years old) and the old old (75–89 years old) following various forms of health shocks. The reasons for the disparities in subjective wellbeing between younger and older persons can be investigated further in future studies by integrating more age groups in the questionnaire data collection.

Finally, mediation analyses assume no uncontrolled confounding between exposure and outcome, exposure and mediator, and mediator and outcome. Validating and supporting these assumptions is difficult in most mediation analyses, including the present study. Although factors that may influence the mediated effect-outcome relationship were controlled for as much as possible in the study model, some uncontrolled confounding may still exist, and possible potential factors can be further identified in subsequent studies, such as through questionnaires or experimental design.

Furthermore, due to the limits of the research data, we were unable to accurately comprehend the frequency of social activity participation and data connected to the study respondents' happiness with social participation. As a result, we were unable to assess the quantity and quality of respondents' social participation in greater depth and discriminate between the social participation of middle-aged people and old persons. The questionnaire method can be utilized in future study to examine differences in the amount and quality of social participation among different groups.

These results also have policy implications. From a service standpoint, the government should improve the development of an integrated medical service system. This will not only promote a more equitable distribution of high-quality medical resources, but will also help to improve the quality of medical services offered by grassroots organizations. In this approach, they may better maintain their health data, provide individualized health recommendations, and prevent the incidence of common and frequent diseases in the middle-aged and older population before they are impacted by health shocks. After middle-aged individuals and older persons have avoided any health shocks, those who have been unwell can receive effective and timely treatment. On the demand side, the government should strengthen the appropriate outpatient medical insurance system, major illness insurance system, and commercial insurance system in order to protect the medical needs of the middle-aged and older adults. Minor illnesses can be easily avoided if middle-aged individuals and older people receive timely outpatient treatment. Improving the major illness insurance system can also ensure that persons in their forties and fifties who have already become unwell receive timely medical treatment. Adopting a healthy lifestyle is critical for people and families to avoid health shocks. This involves eating a well-balanced diet, exercising moderately, getting enough sleep, and avoiding unhealthy behaviors (such as smoking and alcohol misuse). Being socially active is also crucial. Social participation gives support, relieves stress, and increases social capital, all of which contribute to an individual's subjective wellbeing.

## 6 Conclusion

This study aims to evaluate the influence of health shocks on changes in subjective wellbeing among middle-aged individuals and older adults (aged 45+) in China. The study is based on a nationally representative survey, CFPS, utilizing the PSM-DID model. The findings suggest that health shocks can adversely impact the subjective wellbeing of this demographic by affecting their personal relationships. Therefore, interventions aimed at maintaining or elevating the levels of subjective wellbeing among at-risk individuals should be implemented proactively, before the onset of a health shock.

## Data availability statement

Publicly available datasets were analyzed in this study. This data can be found here: http://www.isss.pku.edu.cn/cfps/download.

## Author contributions

CJ, QX, and HG were responsible for literature research and data analysis. Material preparation and data collection were done by HC. The first draft of the manuscript was written by QX, the revision of the literature review and policy recommendations was done by JG, and the data checking and graph organization was done by ZL. All authors commented on a previous version of the manuscript and were involved in the conceptualization and design of the study. All authors read and approved the final manuscript.
