# Supplementary material for: A study on the impact of health shocks on subjective wellbeing of middle-aged people and older adults—Evidence from China
Source: Front Public Health. 2024 Jan 11;11:1238026. doi: 10.3389/fpubh.2023.1238026 (PMC10810137; doi:10.3389/fpubh.2023.1238026)

1 Supplementary Material

Table S1 logit propensity score estimates and balance tests (Health shocks)

Variables	Logit propensity score	Unmatched / Matched	Mean T	Mean C	Bias (%)	Reduct Bias (%)	Two-sample t-test t	p>|t|
Age								
60 years and above	0.462***	U	0.418	0.324	19.7	95.3	5.72	0.000
	(0.094)	M	0.418	0.414	0.90		0.19	0.849
Gender (1=male)	-0.276***	U	0.438	0.524	-17.3	68	-4.90	0.000
	(0.096)	M	0.438	0.412	5.50		1.19	0.235
Education								
Primary school(1=yes)	-0.238**	U	0.256	0.288	-7.2	58.8	-2.02	0.043
	(0.096)	M	0.256	0.269	-3		-0.64	0.522
Lower Secondary(1=yes)	-0.208**	U	0.272	0.302	-6.8	82.1	-1.91	0.056
	(0.099)	M	0.272	0.266	1.2		0.26	0.791
High school and above(1=yes)	-0.156	U	0.119	0.124	-1.4	-307	-0.40	0.690
	(0.135)	M	0.119	0.100	5.7		1.28	0.202
Household registration (1=non-farm)	-0.240**	U	0.143	0.163	-5.5	-0.1	-1.53	0.125
	(0.117)	M	0.143	0.124	5.5		1.24	0.214
Marriage	-0.040	U	0.899	0.919	-6.8	43.5	-2.01	0.044
	(0.122)	M	0.899	0.911	-3.8		-0.80	0.423
Residence	-0.035	U	0.406	0.420	-2.7	-113.5	-0.77	0.439
	(0.079)	M	0.406	0.377	5.8		1.25	0.211
Medical insurance	0.083	U	0.905	0.905	0.1	-7018.2	0.03	0.974
	(0.123)	M	0.905	0.929	-8.3		-1.88	0.061
Pension insurance	-0.021	U	0.462	0.523	-12.2	72.7	-3.45	0.001
	(0.088)	M	0.462	0.479	-3.3		-0.71	0.480
Self-reported economic status	-0.179***	U	2.808	3.070	-24.1	86.5	-6.75	0.000
	(0.038)	M	2.808	2.843	-3.2		-0.70	0.483
Self-reported social status	-0.116***	U	3.089	3.302	-20	97.4	-5.58	0.000
	(0.038)	M	3.089	3.095	-0.5		-0.11	0.912
BMI								
Underweight(1=yes)	0.129	U	0.061	0.046	6.7	-2.3	2.02	0.043
	(0.157)	M	0.061	0.045	6.9		1.47	0.142
Overweight(1=yes)	-0.014	U	0.308	0.326	-3.8	6.1	-1.07	0.285
	(0.081)	M	0.308	0.291	3.6		0.77	0.442
Obese(1=yes)	0.195	U	0.092	0.08	4.1	22.3	1.18	0.238
	(0.128)	M	0.092	0.083	3.2		0.67	0.506
ADL	-0.300***	U	0.830	0.875	-12.8	63.5	-3.84	0.000
	(0.099)	M	0.830	0.847	-4.7		-0.96	0.339
Smoking	-0.045	U	0.385	0.338	-11.5	83.4	-3.21	0.001
	(0.100)	M	0.385	0.276	1.9		0.42	0.676
Drinking	-0.107	U	0.155	0.194	-10.4	32.7	-2.86	0.004
	(0.017)	M	0.155	0.128	7.0		1.62	0.106
Exercise	-0.021	U	0.730	0.723	1.6	-168.7	0.44	0.658
	(0.084)	M	0.730	0.712	4.2		0.89	0.374
Siesta	0.140*	U	0.636	0.601	7.1	64.7	2.00	0.046
	(0.075)	M	0.636	0.624	2.5		0.53	0.593
Internet use	0.181**	U	0.348	0.349	-0.2	100	-0.05	0.957

(0.085)	M	0.348	0.348	0.0	0.00	1.000

Table S2 logit propensity score estimates and balance tests (Chronic health shocks)

Variables	Logit propensity score	Unmatched / Matched	Mean T	Mean C	Bias (%)	Reduct Bias (%)	Two-sample t-test t	p>|t|
Age								
60 years and above	0.482*** (0.113)	U	0.413	0.324	18.5	83.3	4.48	0.000
		M	0.413	0.398	3.1		0.53	0.599
Gender (1=male)	-0.411*** (0.118)	U	0.424	0.524	-20.2	93.4	-4.75	0.000
		M	0.423	0.43	-1.3		-0.23	0.816
Education								
Primary school(1=yes)	-0.243** (0.116)	U	0.248	0.288	-9.2	79.7	-2.13	0.033
		M	0.248	0.256	-1.9		-0.33	0.741
Lower Secondary(1=yes)	-0.106 (0.117)	U	0.290	0.302	-2.6	58.2	-0.61	0.540
		M	0.290	2.860	1.1		0.19	0.849
High school and above(1=yes)	-0.161 (0.164)	U	0.112	0.124	-3.6	85.8	-0.84	0.403
		M	0.111	0.112	-0.5		-0.09	0.927
Household registration (1=non-farm)	-0.299** (0.141)	U	0.139	0.163	-6.9	26.4	-1.59	0.112
		M	0.137	0.119	5.1		0.95	0.344
Marriage	-0.152 (0.140)	U	0.889	0.919	-10.1	55.4	-2.54	0.011
		M	0.889	0.876	4.5		0.71	0.375
Residence	-0.055 (0.095)	U	0.403	0.429	-3.5	-55.7	-0.81	0.415
		M	0.402	0.375	5.4		0.94	0.346
Medical insurance	-0.033 (0.142)	U	0.896	0.905	-2.9	43.0	-0.70	0.485
		M	0.896	0.901	-1.7		-0.29	0.776
Pension insurance	0.024 (0.046)	U	0.472	0.523	-10.2	96.8	-2.43	0.015
		M	0.471	0.469	0.3		0.06	0.954
Self-reported economic status	-0.179*** (0.046)	U	2.789	3.07	-25.8	97.1	-6.03	0.000
		M	2.785	2.793	-0.8		-0.13	0.893
Self-reported social status	-0.138*** (0.046)	U	3.058	3.302	-22.8	86.5	-5.33	0.000
		M	3.055	3.022	3.1		0.53	0.593
BMI								
Underweight(1=yes)	0.009 (0.195)	U	0.054	0.046	4.1	81.3	1.00	0.319
		M	0.055	0.056	-0.8		-0.13	0.900
Overweight(1=yes)	0.020 (0.096)	U	0.317	0.326	-1.9	-51.2	-0.44	0.659
		M	0.316	0.329	-2.8		-0.49	0.623
Obese(1=yes)	0.166 (0.156)	U	0.087	0.08	2.6	-129.1	0.63	0.531
		M	0.088	0.071	6.0		1.06	0.288
ADL	-0.321** (0.119)	U	0.830	0.875	-12.8	89.1	-3.21	0.001
		M	0.830	0.825	1.4		0.23	0.820
Smoking	-0.021 (0.122)	U	0.282	0.338	-12.1	97.0	-2.8	0.005
		M	0.281	0.283	-0.4		-0.06	0.949
Drinking	0.065 (0.125)	U	0.170	0.194	-6.2	-10.5	-1.44	0.150
		M	0.170	0.144	6.9		1.26	0.206
Exercise	-0.096 (0.099)	U	0.717	0.723	-1.4	-465.8	-0.34	0.734
		M	0.716	0.752	-8.1		-1.43	0.153
Siesta	0.141 (0.089)	U	0.635	0.601	7.0	17.3	1.64	0.100
		M	0.636	0.664	-5.8		-1.02	0.306
Internet use	0.192* (0.102)	U	0.351	0.349	0.6	-24.3	0.14	0.885
		M	0.352	0.362	-2.1		-0.36	0.719

Table S3 logit propensity score estimates and balance tests (Acute health shocks)

Variables	Logit propensity score	Unmatched / Matched	Mean T	Mean C	Bias (%)	Reduct Bias (%)	Two-sample t-test t	p>|t|
Age								
60 years and above	0.412*** (0.155)	U	0.43	0.324	22,1	65.6	3.85	0.000
		M	0.43	0.467	-7.6		-0.90	0.367
Gender (1=male)	-0.007 (0.155)	U	0.467	0.524	-11.5	36.4	-1.96	0.050
		M	0.467	0.430	7.3		0.90	0.367
Education								
Primary school(1=yes)	-0.222 (0.157)	U	0.273	0.288	-3.3	77.6	-0.56	0.576
		M	0.273	0.270	0.7		0.09	0.927
Lower Secondary(1=yes)	-0.420** (0.170)	U	0.233	0.302	-15.6	95.2	-2.56	0.011
		M	0.233	0.230	0.8		0.1	0.923
High school and above(1=yes)	-0.147 (0.218)	U	0.133	0.124	2.8	100	0.49	0.624
		M	0.133	0.133	0		0.00	1.000
Household registration (1=non-farm)	-0.144 (0.192)	U	0.153	0.163	-2.7	33.3	-0.46	0.646
		M	0.153	0.147	1.8		0.23	0.819
Marriage	0.214 (0.221)	U	0.920	0.919	0.3	-2798	0.06	0.954
		M	0.920	0.947	-9.8		-1.31	0.191
Residence	0.003 (0.132)	U	0.413	0.420	-1.3	-6	-0.22	0.829
		M	0.413	0.420	-1.4		-0.17	0.869
Medical insurance	0.350 (0.224)	U	0.923	0.905	6.6	10.4	1.08	0.281
		M	0.923	0.907	5.9		0.73	0.465
Pension insurance	-0.124 (0.146)	U	0.443	0.523	-16	95.8	-2.71	0.007
		M	0.443	0.440	0.7		0.08	0.935
Self-reported economic status	-0.179*** (0.063)	U	2.847	3.070	-20.5	47.8	-3.43	0.001
		M	2.847	2.963	-10.7		-1.31	0.191
Self-reported social status	-0.066 (0.064)	U	3.153	3.302	-14.1	86.6	-2.33	0.020
		M	3.153	3.133	1.9		0.24	0.812
BMI								
Underweight(1=yes)	0.335 (0.239)	U	0.073	0.046	11.7	100	2.23	0.026
		M	0.073	0.073	0.0		0.00	1.000
Overweight(1=yes)	-0.081 (0.137)	U	0.290	0.326	-7.7	90.6	-1.29	0.197
		M	0.290	0.287	0.7		0.09	0.928
Obese(1=yes)	0.264 (0.205)	U	0.100	0.080	6.9	100	1.23	0.219
		M	0.100	0.100	0.0		0.00	1.000
ADL	-0.263 (0.205)	U	0.83	0.875	-12.8	48.6	-2.32	0.020
		M	0.83	0.853	-6.6		-0.78	0.435
Smoking	-0.095 (0.163)	U	0.290	0.338	-10.3	44.3	-1.72	0.085
		M	0.290	0.263	5.7		0.73	0.466
Drinking	-0.475** (0.191)	U	0.123	0.194	-19.4	71.7	-3.05	0.002
		M	0.123	0.103	5.5		0.77	0.441
Exercise	0.144 (0.144)	U	0.757	0.723	7.8	-17.4	1.29	0.196
		M	0.757	0.717	9.1		1.11	0.267
Siesta	0.135 (0.124)	U	0.637	0.601	7.3	90.6	1.23	0.221
		M	0.637	0.640	-0.7		-0.08	0.932
Internet use	0.152 (0.144)	U	0.340	0.349	-1.8	61.1	-0.31	0.760
		M	0.340	0.337	0.7		0.09	0.931

Table S4 Results of different types of health shocks interacting with mediating variables

	Health shocks			Chronic health shocks			Acute health shocks		
Treat	-0.182*<br>(0.105)	-0.287***<br>(0.032)	-0.288***<br>(0.032)	-0.007<br>(0.124)	-0.278***<br>(0.038)	-0.286***<br>(0.039)	-0.579**<br>(0.179)	-0.307***<br>(0.052)	-0.291***<br>(0.054)
Partnership	0.194***<br>(0.005)	-	-	0.194***<br>(0.005)	-	-	0.193***<br>(0.005)	-	-
Treat<br>× Partnership	-0.009<br>(0.015)	-	-	-0.033<br>(0.017)	-	-	0.048<br>(0.025)	-	-
Religious belief	-	0.040<br>(0.057)	-	-	0.038<br>(0.057)	-	-	0.039<br>(0.057)	-
Treat<br>× Religious belief	-	-0.118<br>(0.161)	-	-	-0.164<br>(0.184)	-	-	0.011<br>(0.301)	-
Organizational participation	-	-	-0.029<br>(0.034)	-	-	-0.029<br>(0.034)	-	-	-0.035<br>(0.034)
Treat<br>× Organizational participation	-	-	0.023<br>(0.138)	-	-	0.023<br>(0.138)	-	-	-0.196<br>(0.188)
Control variables	Yes								

Fig. S1 Common range of values for propensity scores

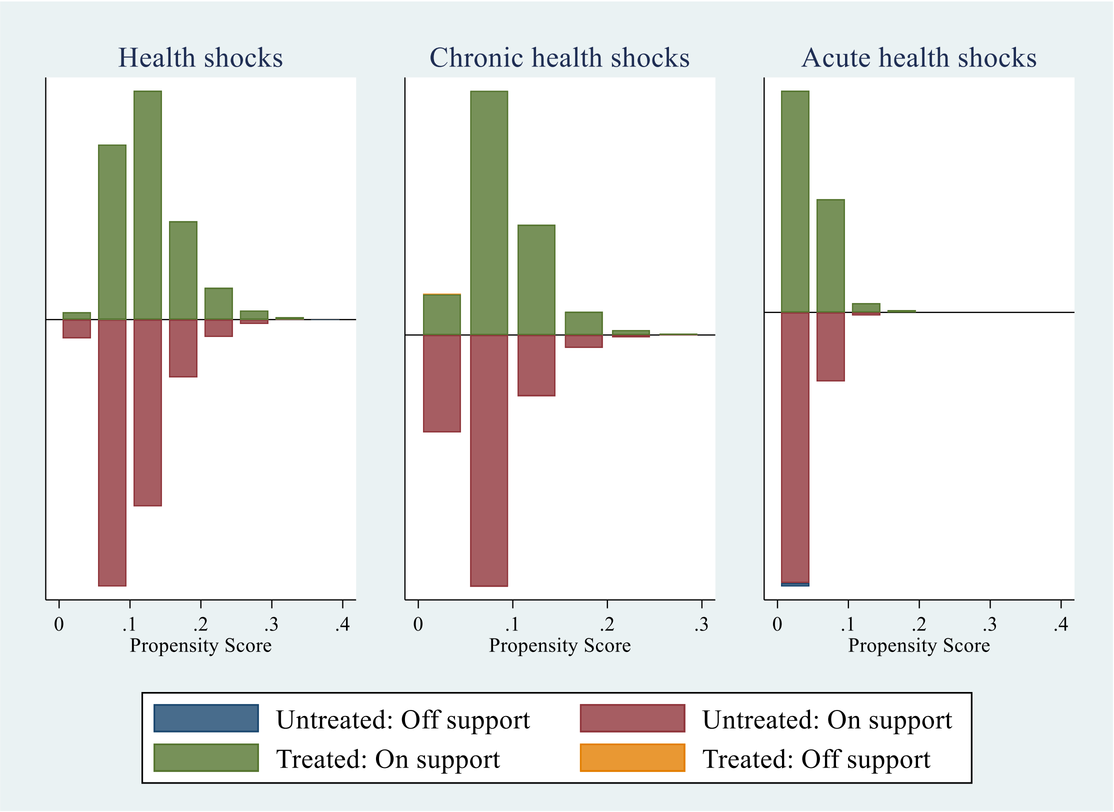

Fig. S2 Kernel density function graph before and after propensity score matching (Health shocks)

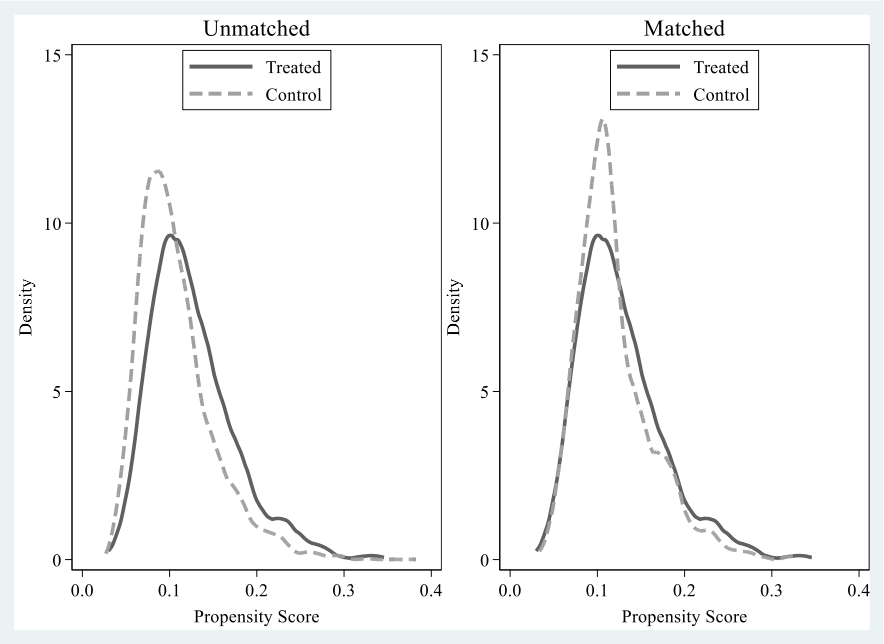

Fig. S3 Kernel density function graph before and after propensity score matching (Chronic health shocks)

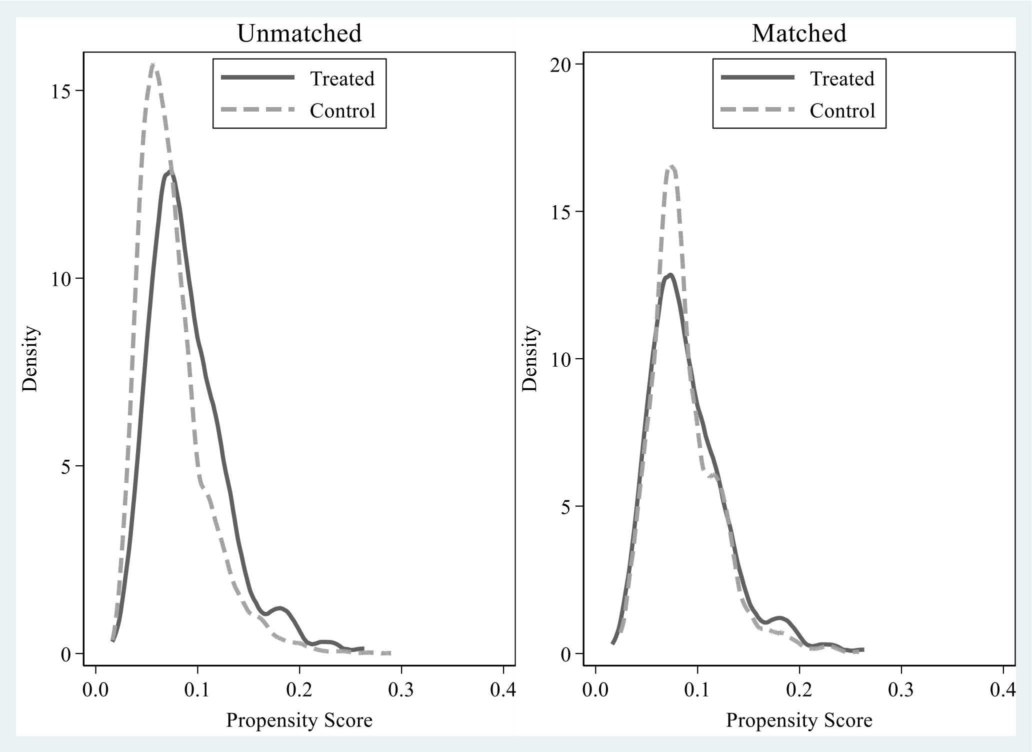

Fig. S4 Kernel density function graph before and after propensity score matching (Acute health shocks)

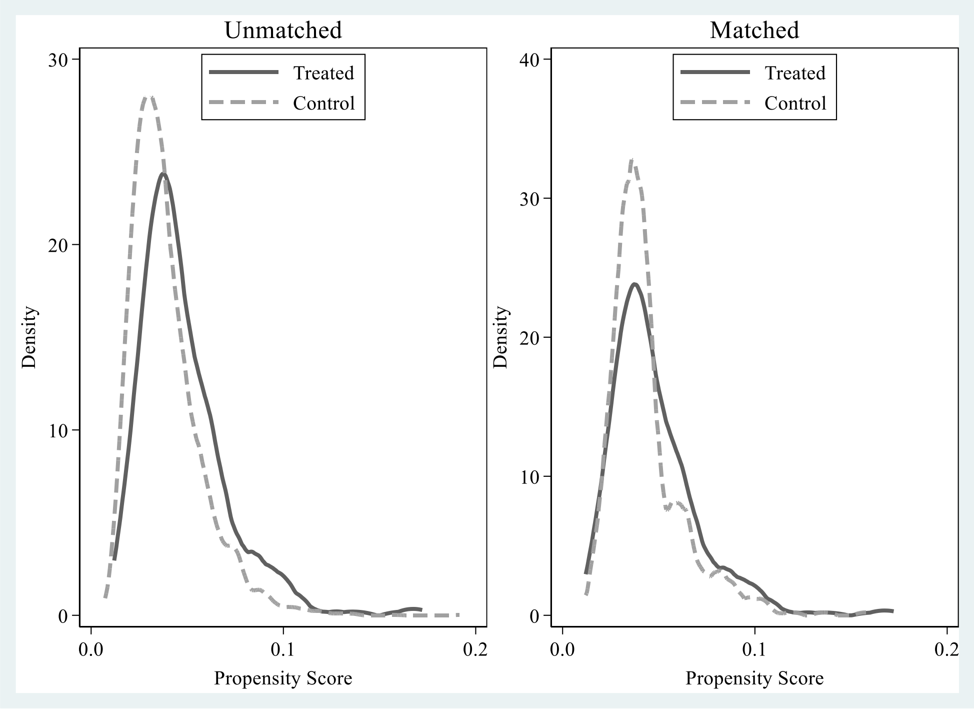

Fig. S5 Placebo test results

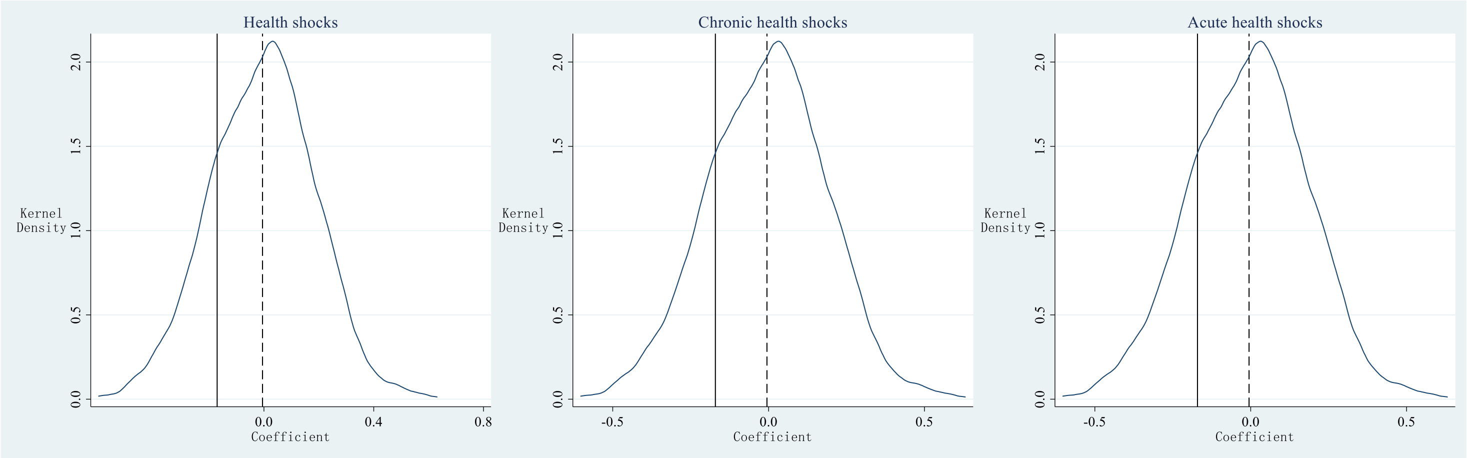

Supplement: Supplementary file 1 [file Data_Sheet_1.pdf]
